# Peter Eckl: Research on the Pro-/Antioxidant Balance

**DOI:** 10.3390/antiox11061079

**Published:** 2022-05-28

**Authors:** Nikolaus Bresgen, Werner Siems

**Affiliations:** 1 Department of Biosciences and Medical Biology, Faculty of Natural and Life Sciences, University of Salzburg, Hellbrunnerstrasse 34, 5020 Salzburg, Austria; 2 Clinics for Prevention and Rehabilitation, 38667 Bad Harzburg, Germany; werner.siems@t-online.de

Peter *Maria* ECKL started his scientific career in the late 1970s at the Paris-Lodron University of Salzburg working in the field of radiation research. For his post-doc, he moved to the lab of Randy Jirtle at the Duke University, Durham (North Carolina, USA) where he succeeded in establishing serum-free culture conditions for proliferating adult primary rat hepatocytes in genotoxicity testing. Returning to Salzburg, Peter ECKL qualified as a professor for “Cytology and Genetics” and in 2015 was appointed full professor at the University of Salzburg.

Since the late 1980s, Peter Eckl intensified his research focus on genotoxicity testing in primary rat hepatocytes and extended the method to fish hepatocytes as a novel approach to aquatic biomonitoring. During this period, the lipid peroxidation products hydroxyalkenals, as was the case for many *HNE-seniors,* also stimulated the interest of Peter Eckl. In this early research, Peter Eckl, in cooperation with the *Grand senior* of lipid peroxidation and 4-Hydroxynonenal (HNE) research, Hermann Esterbauer, from the University of Graz, Austria, investigated the biological effects of HNE, his newly established, serum-free hepatocyte culture system using micronucleus formation, the induction of chromosomal aberrations and sister chromatid exchanges (SCE) as genotoxic endpoints. Based on the initial finding of HNE cyto- and genotoxicity in primary rat hepatocytes [1], his interest focused on the relationship between the genotoxic potential of selected hydroxyalkenals and analogous aldehydes and their chemical composition. This follow-up study, published again in collaboration with Hermann Esterbauer, demonstrated the influence of the length of the lipophilic tail, the presence of the OH group and presence of the CC-double bond on the dose–response and the stimulation of specific genotoxic endpoints of the investigated compounds [2]. Of significance, hepatocytes appeared to be “*at least one order of magnitude more sensitive to the genotoxic action of HNE*” compared to the effects of HNE seen in less metabolically active cells such as V79 fibroblasts [1,3,4]. With this observation, the study emphasized the role of metabolic processing (i.e. Detoxification) in the biological effects of HNE and analogous hydroxyalkenals as well as its potential pathophysiological implications as shown in subsequent research [5,6]. The beginnings of our relationship with Peter Eckl date back to these early years: as one of his students (Nikolaus Bresgen) or as a colleague already working in the field of lipid peroxidation (Werner Siems), and developed into a long–time collaboration and close friendship.

Emerging from this initial research on hydroxyalkenals, the biological aspects of oxidative stress, lipid peroxidation (LPO) and especially “the cell biology of HNE” became an essential focus in Peter Eckl’ s scientific interests, with several lines of investigation converging in this central context. Research conducted in the “Eckl–Lab” in particular comprised studies on the effects of oxidative stress and HNE in cerebral endothelial cells (cECs) and astrocytes. This research revealed a high sensitivity of cECs to HNE, with concentrations ≥ 1µM exerting a significant genotoxic effect [7]. Further experiments using in vitro models for oxidative stress (ischemia/reoxygenation, application of redox-cycling Dimethoxy-1,4-naptoquinone) demonstrated that the genotoxicity exerted by these pro-oxidant regimens in cECs is accompanied by the onset of programmed cell death via apoptosis in a p53-dependent mode which is aggravated by glucose deprivation [8]. Finally, this work showed that astrocytes are less susceptible than cECs to oxidative stress mediated geno- and cytotoxic effects in vitro, which together with our other observations emphasizes the impact of oxidative insults and LPO on blood–brain barrier integrity [9].

During this research period, the “Eckl–Bresgen–Siems collaboration” markedly intensified in another joint project addressing the geno- and cytotoxic effects of β-carotene degradation products. In the 1990s, two large clinical trials, the Alpha-Tocopherol, Beta-Carotene-Cancer-Prevention study (ATCB-study) and the Beta-Carotene and Retinol Efficacy Trial (CARET) investigated the use of dietary high-dose β-carotene supplementation for lowering lung cancer risk, especially in heavy smokers and asbestos workers [10,11]. In contrast to expectations, however, the incidence of lung cancer increased in the β-carotene supplementation cohort of participants (smokers and asbestos workers) forcing the termination of the trials. In addressing this “paradoxon”, Werner Siems and his group could demonstrate that β-carotene cleavage products (CP) comprising aldehydes, carbonyls and epoxides, are formed under conditions of heavy oxidative stress. This also applied to the oxidative degradation of β-carotene by hypochloric acid, simulating the physiologically relevant, non-enzymatic degradation of β-carotene by stimulated polymorph-nuclear leukocytes [12]. Moreover, the oxidative-stress exerted by the CPs themselves adversely affect mitochondrial function in isolated liver mitochondria [13] and we were interested in whether CPs promote toxic effects in primary rat hepatocytes as well as primary type II rat pneumocytes. These experiments demonstrated significant genotoxic properties for CPs in the physiologically relevant micromolar range (1–10 µM) in both cell types, which were aggravated by pro-oxidant regimens [14,15]. Moreover, application of β-carotene under oxidative stress conditions also stimulated genotoxic events in hepatocytes but not in pneumocytes, a finding that emphasizes the role of oxidative β-carotene degradation as a promotor of carcinogenesis and supposedly reflects differences in β-carotene metabolism and sensitivity to β-carotene degradation products between different cell types under conditions of oxidative stress [16,17].

To revisit “HNE biology”, the observation that serum HNE-levels increase with age (reviewed in [6]) called our attention and we continued our collaboration by addressing age-related aspects of HNE degradation in vitro. Using fibroblast cultures established from human skin samples, we observed that HNE metabolism declines with donor age (Figure 1). A follow-up investigation conducted by Petkovic et al. [18], which is presented in this Special Issue of ‘Antioxidants’, confirmed the observed age dependence of HNE degradation and revealed that fibroblasts from older donors show obviously lower GSH levels which limits HNE detoxification via the formation of GSH-HNE conjugates.

The observed association between reduced HNE degradation and increased serum HNE levels with aging places emphasis on the implication of LPO and its fatty-acid derived breakdown products in the development of degenerative diseases, especially of the nervous system [20]. In addition, dietary factors stimulating LPO may also play an important role in disease development. As an example, this holds true for the stimulation of intestinal LPO upon consumption of heme/iron-rich red meat which is considered as a considerable risk factor for the development of colorectal cancer (CRC), one of the most frequent and deadly malignancies worldwide today [21]. With high relevance to this, in this Special Issue, Chevolleau et al. [22] present a novel approach for screening diet-related carbonyls as a promising method for tracking the presence of LPO-derived hydroxyalkenals in feces (fecal water), a condition which is considered to promote CRC tumorigenesis. Notably, addressing a similar nutrition-associated context, Peter Eckl in his early work on the usability of primary rat hepatocyte cultures for genotoxicity testing observed that the dietary uptake of mutagens/carcinogens by experimental animals influenced the background incidence of DNA damage (sister chromatid exchanges) seen in the cultured hepatocytes [23,24]. Moreover, follow-up experiments demonstrated that the supplementation of the culture medium with vitamin C and E lowers the genotoxic background (micronucleus formation) seen in primary hepatocyte cultures by about 50%, a finding which emphasized the impact of oxidative stress existing in vitro on cultured cells [25].

Dating back to the late 1980s, these observations fit quite well in the body of evidence existing today for the pivotal role of cellular antioxidant defenses in counterbalancing the adverse effects of pro-oxidant conditions. Thus, it is not surprising that publications on “antioxidants”, within the last 80 years, yielded a total number of nearly 670,000 publications listed in PubMed today (Table 1). In this commemorative issue, three articles directly address antioxidant mechanisms. Sommerburg et al. [26] report changes in plasma vitamin A and E levels in cystic fibrosis patients under lumacaftor-ivacaftor therapy. The report emphasizes that the shift of plasma vitamin A (retinol) bears the risk of hypervitaminosis-driven consequences, in particular hepatotoxicity, in these patients. In another report, Altomare et al. demonstrate that the antioxidant activity of N-Acetyl-Cysteine is conferred by an albumin Cys34 regenerating mechanism, which improves the plasma antioxidant potential [27]. Additionally, related to oxidative stress-related cysteine modifications, the paper by Mónico et al. [28] discusses the identification of a “hot-spot” for zinc-binding on the intermediate filament protein vimentin at Cys328, a finding which provides novel insights into stress-associated protein modifications in the context of cellular redox state and zinc metabolism.

Complementary to the elucidation of pro-/antioxidant mechanisms, investigating natural as well as synthetic compounds for pro-/antioxidant properties with relevance to health contexts is also the subject of intensive biological and pharmacological research. This also holds true for our research on β-carotene as addressed above. Moreover, our interests expanded in collaboration with several other laboratories by investigating antioxidant, radioprotective properties of selected compounds such as glucans [37] and in particular, by conducting ethno-pharmacological research examining the cyto- and genotoxic properties of natural, herbal compounds used in traditional medicine. Among several of our studies addressing herbs used, for example, in Chinese and Mongolian traditional medicine, the cytogenetic analysis of extracts from Palestinian medicinal plants provided interesting insights into the pharmacological/toxicological assessment of herbal compounds [38]. In particular, a study on thymoquinone, the main constituent of essential oil prepared from *Nigella sativa* (black seed) which is widely used in Arab herbal medicine [39], emphasized the impact of hepatic biotransformation in defining the anti-/promutagenic potential of such compounds [40].

Parallel to this, with the finding of ferritin cytotoxicity in 2002, iron elicited our scientific interest [41]. Although not unexpected, this turned out to be yet another “lipid-peroxidation/HNE associated story” [42,43] which from today’s point of view adheres well to the rapidly emerging concept of *ferroptosis*—essentially a LPO-based mode of necrotic cell death. Our research on ferritin-mediated cell death revealed that both apoptosis and necrosis result from a complex interplay between the cellular labile (i.e. non-bound) iron pool—especially inside the endo-/lysosomal compartment, LPO and in terms of glutathione availability, the autophagic flux and lysosomal function [43]. As a remarkable feature occurring under the iron-dependent, pro-oxidant regimen, we observed the intracellular accumulation of HNE-modified proteins, also inside autophagosomes, which suggested a strategy of hepatocytes to sequester and detoxify HNE-modified proteins by autophagy. Korovila et al. [44], in their contribution to this commemorative Special Issue, present findings on liver autophagy using a mouse model for obesity. The authors show that feeding these animals a high-fat diet stimulates the biogenesis of lipid droplets accompanied by a reduced autophagic activity with a maintained 20S proteasome function and the hepatic accumulation of HNE-modified proteins; findings which are in good correspondence to ours and place an emphasis on the emerging role of autophagy in the pro-/antioxidant interplay affecting proteostasis under stress conditions.

Gifted with a generous, considerate and creative personality, Peter Eckl was a highly respected colleague, teacher and discussion partner, especially in discussions on new aspects of research and new hypotheses. Peter ECKL also was an engaged scientific networker. He participated in numerous national and international research activities and scientific organizations. As one of the founders of the HNE-Club, Peter Eckl was always strongly affiliated with the constantly expanding community of researchers working on LPO and HNE for which he served many years as chair. Unexpectedly, Peter ECKL passed away on 28 September 2019. With him, the scientific community lost a merited, esteemed member, a “convinced HNE-ambassador” and for many an outstanding and valuable friend (Figure 2).

## Figures and Tables

**Figure 1:**
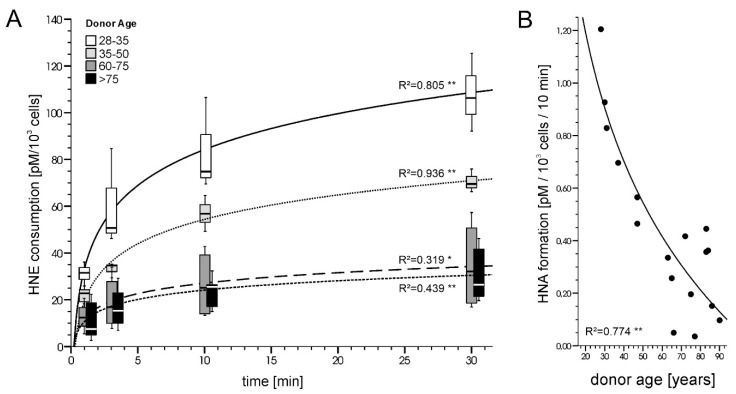
Age dependence of HNE metabolism in cultured human skin fibroblasts. Cell cultures were established from donors of different ages and treated with 1 µM HNE. (**A**) HNE consumption curves (expressed as pM HNE per 10^3^ cells) determined 1, 3, 10 and 30 min after HNE addition. Data of the age cohorts are presented as Box-Whisker Plots. (**B**) Formation of hydroxynoneoic-acid (HNA) from HNE in the fibroblast cultures expressed as pM HNA/10^3^ cells/10 min). * *p* < 0.05; ** *p* < 0.005 significance of the correlation (Pearson): [Siems W., Voss P., Bresgen N., Eckl P. and Grune T., unpublished data] [19].

**Figure 2:**
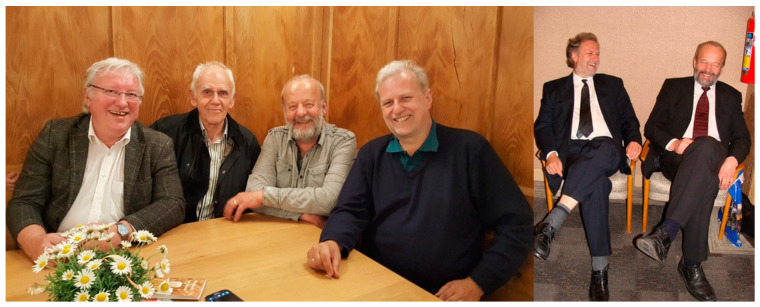
Nikolaus Bresgen and Werner Siems, Mai 2022. **Left.** Werner Siems, Rudolf Jörg Schaur (a close collaborator of Hermann Esterbauer), Peter Eckl and Nikolaus Bresgen (from left) debriefing their joint lecture series on “Oxidative stress and lipid peroxidation” at the University of Salzburg. **Right.** Peter Eckl and Nikolaus Bresgen—close collaborators for more than 25 years-in a typical conversation at the 4th international meeting of the HNE-Club 2008 in Karuizawa, Japan.

**Table 1:** Publication metrics on selected terms Listed in the PubMed database.

	Year of First Entry	Entries in Year 1987 ^1^	Entries in Year 2021	Total Entries ^2^
Antioxidants	1942 *	4181	41,666	669,369
Oxidative stress	1960 **	95	28,257	280,672
Lipid peroxidation	1958	578	3889	79,687
4-Hydroxynonenal ^3^	1980 ^4^	19	281	5664

^1^ The year 1987 falls into the time period when research on antioxidants began to increase substantially (according to PubMed metrics). ^2^ The total number of publications listed in PubMed which accumulated between the first publication and the most recent entry on 17 May 2022. ^3^ Performing the search using the term ‘HNE’ returns 4505 entries (total) since 1982 and using the more precise term ‘4-Hydroxy-2-nonenal’ returns 3967 publications (total) starting in 1980. ^4^ Among the first entries in 1980, Benedetti A., Comporti M. and Esterbauer H. for the first time report cytotoxic effects of HNE [29]. However, assumedly representing the first evidence for biological/cytotoxic effects of 4-hydroxy-alkenals, Schauenstein E, Wünschemann B., and Esterbauer *H.* in 1968 [30] reported the “in vivo *destruction of subcutaneously implanted Ehrlich ascites tumor cells using 4-hydroxy-pentenal*”.* Already the first entry listed in Pubmed under the term “antioxidants” from György et al., 1942 [31] anticipates the context of LPO-based disease and antioxidant counteraction. This publication reports that feeding rats a special diet ‘By’ (which stands for “butter yellow”) containing linoleic acid supplemented with p-dimethylaminoazobenzene (p-DAB) proved to stimulate severe pathologies (weight loss, progressive anemia, leucopenia and pediculosis), which was enhanced when the DAB was omitted (!) from the diet. Highlighting the impact of antioxidant defenses, adding cystine and choline to this linoleic acid-/DAB-supplemented diet prolonged the survival of the experimental animals. Moreover, in a further experiment, rats were fed a diet of linoleic acid stored for several weeks in the presence of DAB under room temperature and the authors report, that the “*linoleic acid was almost completely oxidized and destroyed by keeping diet “By,” before use, for 3 to 4 weeks at laboratory temperature, and the diet then exerted marked toxic effect on all 23 rats in this group*”. In discussing their findings, the authors come to a striking conclusion: “*It can be assumed that these unsaturated fatty acids, perhaps through formation of peroxides, decompose butter yellow and at the same time give rise to formation of toxic by-products”* [György at al., 1942. J Exp Med 76 (5), p.418] [31]. ** For 1960–1990, PubMed lists almost 1000 publications on the search term “oxidative stress”, the first paper using the term in the title itself being published in 1970 [32]. The same year, Sies and Chance, 1970 [33] published a key-paper demonstrating for the first time the steady-state of endogenous H_2_O_2_ in perfused rat liver. In continuation, Helmut Sies coined the term “oxidative stress” in the mid-1980s as an integrative concept emerging from the thitherto-accumulating research in redox biology [34,35,36], extending the *eustress-distress concept* defined by Hans Selye 50 years earlier to cellular stress physiology. Starting within the mid to late 1980s, research activity in oxidative stress-related topics intensified markedly, the number of publications on “oxidative stress” exceeding 1000 publications per year in 1995 (1125 entries). It appears noteworthy to mention here, that Helmut Sies in 1977 together with his colleagues working in Munich (Germany) on oxygen-related topics also founded the “Münchner Sauerstoffclub” (Munich Oxygen Club) [34], an ancestor of many today’s scientific “clubs” dedicated to oxidative stress. This also holds true for the “HNE-club”, founded in late 2000 with Peter Eckl serving as a founding member and year’s long coordinator.
